# Qualitative comparative analysis of the implementation fidelity of a workplace sedentary reduction intervention

**DOI:** 10.1186/s12889-022-13476-3

**Published:** 2022-05-31

**Authors:** Krista S. Leonard, Sarah L. Mullane, Caitlin A. Golden, Sarah A. Rydell, Nathan R. Mitchell, Alexis Koskan, Paul A. Estabrooks, Mark A. Pereira, Matthew P. Buman

**Affiliations:** 1 College of Health Solutions, Arizona State University, 425 N 5 th Street, Phoenix, AZ 85004 USA; 2 Johnson & Johnson Health and Wellness Solutions, Inc, New Brunswick, USA; 3 College of Public Health, University of Nebraska Medical School, Omaha, USA; 4 School of Public Health, University of Minnesota, Minneapolis, USA; 5 College of Health, University of Utah, Salt Lake City, USA

**Keywords:** Workplace, Sedentary, Implementation, Fidelity, Adherence, Competence

## Abstract

**Background:**

*Stand and Move at Work* was a 12-month, multicomponent, peer-led (intervention delivery personnel) worksite intervention to reduce sedentary time. Although successful, the magnitude of reduced sedentary time varied by intervention worksite. The purpose of this study was to use a qualitative comparative analysis approach to examine potential explanatory factors that could distinguish higher from lower performing worksites based on reduced sedentary time.

**Methods:**

We assessed 12-month changes in employee sedentary time objectively using accelerometers at 12 worksites. We ranked worksites based on the magnitude of change in sedentary time and categorized sites as higher vs. lower performing. Guided by the integrated-Promoting Action on Research Implementation in Health Services framework, we created an indicator of intervention fidelity related to adherence to the protocol and competence of intervention delivery personnel (i.e., implementer). We then gathered information from employee interviews and surveys as well as delivery personnel surveys*.* These data were aggregated, entered into a truth table (i.e., a table containing implementation construct presence or absence), and used to examine differences between higher and lower performing worksites.

**Results:**

There were substantive differences in the magnitude of change in sedentary time between higher (-75.2 min/8 h workday, CI_95_: -93.7, -56.7) and lower (-30.3 min/8 h workday, CI_95_: -38.3, -22.7) performing worksites. Conditions that were present in all higher performing sites included implementation of indoor/outdoor walking route accessibility, completion of delivery personnel surveys, and worksite culture supporting breaks (i.e., adherence to protocol). A similar pattern was found for implementer willingness to continue role and employees using face-to-face interaction/stair strategies (i.e., delivery personnel competence). However, each of these factors were also present in some of the lower performing sites suggesting we were unable to identify sufficient conditions to predict program success.

**Conclusions:**

Higher intervention adherence and implementer competence is necessary for greater program success. These findings illustrate the need for future research to identify what factors may influence intervention fidelity, and in turn, effectiveness.

**Trial registration:**

ClinicalTrials.gov Identifier: NCT02566317. Registered 2 October 2015, first participant enrolled 11 January 2016.

**Supplementary Information:**

The online version contains supplementary material available at 10.1186/s12889-022-13476-3.

## Background

Employee wellness programs strive to promote a healthy lifestyle for employees, maintain or improve health and wellbeing, and prevent or delay the onset of disease [1]. Individuals spend up to 60% of their waking hours in their workplace, making this a highly opportune setting for health promotion programs [2]. Worksite wellness programs typically assess participants’ health risks and deliver tailored educational and lifestyle management interventions designed to lower risks and improve health outcomes [1]. More recently, sedentary time (i.e., waking behaviors in a seated or reclining posture at < 1.5 metabolic equivalents [METs]) [3] has been recognized as a unique health risk factor for cardiometabolic diseases and early mortality [4–8]. American adults currently spend > 7.5 h/day being sedentary, and desk-based workers are at particular risk as they spend 70–90% of their workday sitting at a desk [9]. The workplace, therefore, poses a complex challenge, providing both an environment conducive to promoting undesirable behaviors (i.e., sedentary time), while also posing a highly opportune setting for implementing change. Therefore, workplace interventions to reduce sedentary time have emerged as an important public health priority [10–13].

There is growing impetus to maximize the effectiveness of evidence-based worksite wellness programs [14–16]. However, worksites are not homogenous environments, and translating evidence-based interventions into practice is challenging [17]. For example, worksites may not have the required resources to run an intervention, and the intervention may not be supported by the culture of the organization. This may, in turn, negatively impact intervention implementation fidelity and effectiveness [18]. Implementation fidelity, defined as the extent to which an intervention or program is delivered as intended, is critical to the successful translation of evidence-based interventions into practice [19–21]. If delivered with poor fidelity, evidence-based programs may not have the anticipated health and societal impact [22].

Implementation fidelity can be assessed in several ways [19]; however, the most common way to measure fidelity is by assessing adherence to the intervention protocol [23, 24]. More recently, implementation researchers have proposed operationalizing fidelity as the product of adherence to a specific intervention protocol and competence or quality of delivery of the personnel implementing the protocol [25]. This conceptualization of fidelity is highly relevant to worksite wellness programs given their reliance on existing workplace staff whose experience and training to deliver health programs may vary. Variations across sectors and organizational structures (e.g., allowing for breaks) may also influence program fidelity [17]. Assessing intervention adherence, competence of those who carry out the program, and the context or environment in which the intervention was delivered is necessary to advance our understanding of implementation fidelity and, ultimately, design effective and sustainable workplace interventions [25, 26]. Unfortunately, recent largescale, multi-component workplace interventions which have aimed to reduce sedentary time have reported limited or no fidelity data specific to the intervention components [10, 11].

The *Stand and Move at Work (SMW)* trial tested two multi-component, behavioral interventions to reduce sedentary time in the workplace. The *STAND* + and *MOVE* + interventions were both drawn from the social ecological model targeting the individual, social environment, physical environment, and workplace policies. The *STAND* + intervention included a sit-stand workstation whereas the *MOVE* + intervention did not [12]. The *STAND* + intervention was effective in reducing sedentary time, however, these reductions varied by site, potentially as a result of different implementation patterns [13]. The purpose of this study was to examine these variations in intervention outcomes in relation to differences in implementation fidelity, defined as both adherence to the protocol and competence of the worksite advocates who delivered the program [19, 25]. A secondary purpose was to identify potential factors that could generalize to higher quality implementation, if applied to worksite health promotion initiatives. We hypothesized that implementation fidelity varied across worksites and that the variability would be related to variations in sedentary time.

## Methods

### Overview

To better understand implementation fidelity and potential factors that could either promote or impede implementation, we used the integrated-Promoting Action on Research Implementation in Health Services (i-PARIHS) framework as our conceptual model [27]. The i-PARIHS framework provides a conceptual approach to operationalize implementation fidelity and potential determinants of high-quality implementation. This includes a focus on characteristics of the intervention or innovation (i.e., *SMW*) being tested for effectiveness, the context (i.e., worksite leadership, culture) within which the intervention is being implemented, the facilitation strategies used to promote implementation quality, and the characteristics and actions of recipients (i.e., advocates), or those responsible for implementation [27]. Within i-PARIHS, successful implementation is operationalized as the achievement of implementation goals. The i-PARIHS framework was chosen as our conceptual framework given our interest in better understanding implementation as a result from the facilitation of the innovation (i.e., *SMW*) with the recipients (i.e., advocates) in their context (i.e., worksite culture) [27].

Understanding the context under which an intervention works and how variations in implementing an intervention may lead to successful outcomes is essential for translating evidence-based programs into diverse settings [19–21]. Translational research is often conducted with small samples sizes and lack adequate power to support conventional statistical analyses. Methods like qualitative comparative analysis (QCA) combine quantitative and qualitative techniques among small sample sizes to understand the necessary (i.e., conditions present in all of the higher-performing worksites and some of the lower-performing worksites; high performance will not occur in the absence of these conditions) and sufficient (i.e., conditions present in all of the higher-performing worksites and none of the lower-performing worksites; high performance always occurs in the presence of these conditions) factors that may serve as causal pathways to a desired outcome. QCA is an analytic approach informed by set-theoretical assumptions that allows for systematic cross-case comparisons across a small number of cases. QCA works under the premise that a single outcome may occur due to different causal conditions or a combination of conditions (i.e., variables or determinants) by focusing on commonalities across cases and the association of those commonalities with the outcome [28–31]. QCA has successfully been used across different contexts, including studies conducted in workplaces [32–35]. We used QCA to explore the variation in the implementation outcomes (i.e., adherence and competence) of *SMW* as it relates to the magnitude of change in sedentary time (i.e., effectiveness).

### Participants

We recruited worksites from the Phoenix, AZ and Minneapolis/St. Paul, MN, USA greater metropolitan regions and selected worksites using purposive sampling across academic, industry/healthcare, and government sectors. We contacted worksites by email and telephone and provided them with brief informational handouts detailing study goals and expectations. Full details of recruitment strategies are published elsewhere [12].

We enrolled worksites to participate in *SMW* if they met the following inclusion criteria: (a) had a small to moderate workgroup size (i.e., 20–60 employees); (b) > 80% of employees worked full time; (c) daily work activities involved predominantly seated desk-based office work; (d) not currently participating in a worksite wellness program to reduce sedentary time or increase light-intensity physical activity (LPA); (e) < 10% of employees using a sit-stand workstation; (f) willing to have sit-stand workstations installed; and (g) leadership was willing to be randomized to either study arm. Employees within the worksites were eligible if they were (a) 18 years or older; (b) in generally good health and able to safely reduce sedentary time and increase LPA; (c) working full-time on-site; (d) not currently pregnant; (e) working in an occupation requiring seated office work; (f) not currently using a sit-stand workstation; (g) willing to have a sit-stand workstation installed at their desk; and (h) willing to be randomized to either study arm. Employees completed screening via questionnaire followed by in-person adjudication.

### Study design

A full description of the intervention arms are reported elsewhere [12, 13]. In short, *N* = 24 worksites (630 employees) were randomized to either the *MOVE* + (*N* = 12 worksites) or *STAND* + (*N* = 12 worksites) 12-month intervention. The multi-component interventions consisted of a manualized toolkit grounded in a social-ecological, multi-level framework. All worksites were provided with the toolkit to facilitate behavior change at the organizational, environmental, social, and individual level, designed to cooperatively impact worksite culture. The toolkit presented a menu of strategies to encourage employees to reduce sedentary time during the workday. Of these strategies, 10 were required (i.e., strategies that were required to be implemented by advocates during the intervention period) for participation [12]. The goal of the *MOVE* + intervention was to increase intervention site participants’ light-intensity physical activity by 30 min throughout the workday. In addition to the multi-component intervention, recipients in the *STAND* + intervention received sit-stand workstations with a goal to both increase physical activity by 30 min per workday and increase standing time to 50% of desk-based worktime [12]. We examined differences in intervention implementation using the i-PARIHS framework across the 12 *STAND* + worksites, the more effective intervention arm, to inform future dissemination efforts.

### Measures

#### Effectiveness outcomes

The primary outcome was 12-month change in worksite sedentary time, objectively measured using the activPAL3 micro accelerometer (PAL Technologies, Glasgow, Scotland), the gold standard field-based measure of sedentary time [36]. For this study, sedentary time was defined as sitting with low energy expenditure. Employees were asked to wear the activPAL device for seven consecutive days at baseline, 3-months, 12-months, and 24-months. Worksite sedentary time was standardized to an 8 h workday (i.e., standardized min = observed min × 480/observed min of wear time). Change in 12-month worksite sedentary time was calculated as sedentary time at 12-months minus sedentary time at baseline. The *STAND* + intervention arm demonstrated significantly more reductions in sedentary time compared to the *MOVE* + worksites [13].

#### Implementation fidelity

We assessed adherence to the toolkit and competence of worksite advocates using multiple methods (e.g., self-report surveys, observation, objective survey and web analytics) and gauging various perspectives (e.g., advocates, employees, and study staff; see Table 1 and Supplementary File 1). This resulted in *n* = 190 employee surveys and *n* = 21 “advocate” (champions of the program) surveys at 12-months. Implementation fidelity outcomes were averaged to represent a worksite value when there were multiple individuals (e.g., two advocates from the same worksite or completed the survey) completing the same method.

**Table 1:** Study indicators of i-PARiHS constructs and related decision rules to distinguish between presence or absence of these indicators

i-PARIHS Construct	Condition	Decision Rule
Innovation	Indoor walking route accessibility^a,d^	Scores < 3 were considered not accessible (never or rarely), scores ≥ 3 were considered accessible (sometimes, most of the time, or always)
	Indoor walking route signage visibility^a,d^	Scores < 3 were considered not visible (never or rarely), scores ≥ 3 were considered visible (sometimes, most of the time, or always)
	Outdoor walking route accessibility^a,d^	Scores < 3 were considered not accessible (never or rarely), scores ≥ 3 were considered accessible (sometimes, most of the time, or always)
	Outdoor walking route signage visibility^a,d^	Scores < 3 were considered not visible (never or rarely), scores ≥ 3 were considered visible (sometimes, most of the time, or always)
	Communal signage visibility^a,d^	Scores < 3 were considered not visible (never or rarely), scores ≥ 3 were considered visible (sometimes, most of the time, or always)
	Individual signage visibility^a,d^	Scores < 3 were considered not visible (never or rarely), scores ≥ 3 were considered visible (sometimes, most of the time, or always)
	Stair signage visibility^a,d^	Scores < 3 were considered not visible (never or rarely), scores ≥ 3 were considered visible (sometimes, most of the time, or always)
	Optional cultural strategies chosen^a,d^	Percents < 50 were considered limited optional strategies chosen, percents ≥ 50 were considered moderate-high optional strategies chosen
	Optional environmental strategies chosen^a,d^	Percents < 50 were considered limited optional strategies chosen, percents ≥ 50 were considered moderate-high optional strategies chosen
	Optional social strategies chosen^a,d^	Percents < 50 were considered limited optional strategies chosen, percents ≥ 50 were considered moderate-high optional strategies chosen
	Sent e-newsletters^c,d^	Percents < 80 were considered limited e-newsletters sent, percents ≥ 80 were considered most e-newsletters sent
	Supported informal hourly breaks^c,d^	Percents < 80 were considered limited support for hourly breaks, percents ≥ 80 were considered high support for hourly breaks
	Completed quarterly meeting^c,d^	Percents < 80 were considered limited completion of quarterly meetings, percents ≥ 80 were considered high completion of quarterly meetings
	Completed advocate survey^c,d^	Percents < 80 were considered limited completion of advocate survey, percents ≥ 80 were considered high completion of advocate survey
	Supported email distribution^c,d^	Percents < 80 were considered limited support for email distribution, percents ≥ 80 were considered high support for email distribution
	Completed community readiness interview^c,d^	Percents < 80 were considered limited completion of community readiness interview, percents ≥ 80 were considered high completion of community readiness interview
Context	Worksite culture supported breaks^b,d^	Percents < 80 were considered limited support for breaks, percents ≥ 80 were considered high support for breaks
	Worksite leadership supported breaks^b,d^	Percents < 80 were considered limited support for breaks, percents ≥ 80 were considered high support for breaks
	Months used desk of total^b,d^	Scores < 3 were considered low use, scores ≥ 3 were considered high use
	Perceived morale for the program^b,e^	Scores < 4 were considered as low perceived morale, scores = 4 were considered neutral, and scores > 4 were considered as high perceived morale
	Existing Efforts^b,f^	Scores < 5 were considered low existing community efforts, scores ≥ 5 were considered some existing community efforts
	Knowledge of Efforts^b,f^	Scores < 5 were considered low knowledge of existing community efforts, scores ≥ 5 were considered high knowledge existing community efforts
	Leadership^b,f^	Scores < 5 were considered low leadership recognition/efforts, scores ≥ 5 were considered high leadership recognition/efforts
	Climate^b,f^	Scores < 5 were considered negative community climate, scores ≥ 5 were considered positive community climate
	Knowledge About Issue^b,f^	Scores < 5 were considered low knowledge about the issue, scores ≥ 5 were considered high knowledge about the issue
	Resources^b,f^	Scores < 5 were considered low resources available for the issue, scores ≥ 5 were considered high resources available for the issue
	Overall community readiness score^b,f^	Scores < 5 were considered low community readiness, scores ≥ 5 were considered high community readiness
Recipient	Advocate's interaction with employees^a,e^	Percents < 50 were considered limited advocate-employee interactions, scores ≥ 50 were considered moderate-high advocate-employee interactions
	Knowledge of employees^a,e^	Percents < 50 were considered advocate having limited knowledge of employees, scores ≥ 50 were considered advocate having moderate-high knowledge of employees
	Advocates self-efficacy in role^a,e^	Percents < 50 were considered low self-efficacy, scores ≥ 50 were considered high self-efficacy
	Advocate's willingness to continue role^a,e^	Scores < 3 were considered low willingness of advocate to continue their role, scores ≥ 3 were considered high willingness of advocate to continue their role
	Time spent in the last quarter^a,e^	Scores < 5 were considered less time spent in advocate role, scores ≥ 5 were considered more time spent in advocate role
	Time willing to spend in role next quarter^a,e^	Scores < 5 were considered less time willing to spend in advocate role, scores ≥ 5 were considered more time willing to spend in advocate role
	Employees aware of advocate^b,e^	Percents < 50 were considered low awareness, percents ≥ 50 were considered moderate-high awareness
	Removed wastebin^b,e^	No = wastebin was not removed from office area, yes = wastebin was removed from office area
	Removed printer^b,e^	No = printer was not removed from office area, yes = printer was removed from office area
	Stood in a meeting^b,e^	No = did not stand in a meeting, yes = stood in a meeting
	Walked in a meeting^b,e^	No = did not walk in a meeting, yes = walked in a meeting
	Used face-to-face interaction^b,e^	No = did not use face-to-face interaction, yes = used face-to-face interaction
	Used the stairs^b,e^	No = did not use stairs, yes = used stairs
	Attended at least one group advocate call^c,e^	No = did not attend any group advocate calls, yes = attended at least one group advocate call

^a^indicates data obtained from advocate perspective;

^b^indicates data obtained from employee perspective;

^c^indicates data obtained from researcher observation;

^d^indicates construct for adherence;

^e^indicates construct for competence;

^f^indicates construct for community readiness

##### Adherence

We calculated intervention adherence using a checklist of required intervention activities. We assessed adherence to required environmental components from four online (via Qualtrics [Salt Lake City, UT]) audit surveys sent quarterly to all advocates at each worksite: We asked advocates to rate the accessibility and visibility of the walking routes and signage on a Likert scale (e.g., not accessible at all [1] to highly accessible [5]), and to report the optional items their program advocate selected each quarter. The program required worksites to select at least one optional environmental (*n* = 4), cultural (*n* = 8) or social (*n* = 3) strategy to employ over the 12-month intervention period. We assessed the required cultural components (e.g., promoting a culture that supported hourly informal desk breaks, ensuring leadership were supportive of hourly desk breaks, and openly communicating and advocating for sit-stand workstation use) with three questions. We measured adherence to required cultural components using a 12-month evaluation survey sent via Qualtrics to all employees. We asked employees whether they felt the culture and leadership were supportive of informal hourly desk breaks (Yes/No) and to indicate how much they used the sit-stand workstation over the 12-month period (1 = 0–4 months, 2 = 5–8 months, 3 = 9–11 months, 4 = 12 months). Required organizational components included the following items: allowing the distribution of 26 e-newsletters, allowing informal hourly breaks, advocate participation in quarterly meetings with the researcher, completion of quarterly advocate surveys, program support emails sent by organizational managers, and completion of employee community readiness interviews. Adherence to organizational components was measured using researcher-derived observation (in-person quarterly meetings and web analytics to assess survey completion).

##### Competence

Workplace advocates were considered the “key facilitators” [27] of the program, and they were responsible for delivering and championing the program. Therefore, we calculated competence (i.e., quality of delivery) by using tools that evaluated the efforts of the workplace advocates. We asked advocates to complete a quarterly survey, reporting their employee interaction and knowledge, perceived self-efficacy in carrying out the program, and time and willingness to continue serving as worksite leaders for this program. We asked intervention worksite employees to complete study surveys which assessed the awareness of their colleague as an intervention advocate and perceived worksite support for the program. The employee main study survey (sent via Qualtrics at baseline, 3 m and 12 months) allowed employees to report whether they engaged in a toolkit related behavior in the past month (i.e., removed their waste bin, removed printer, stood in a meeting, walked in a meeting, used face-to-face interactions, used the stairs) to assess whether the advocate’s selected program strategy resulted in intervention worksite employee behavior change. Finally, we used researcher-observed attendance records to assess advocates’ attendance in optional monthly group calls for study advocates.

##### Fidelity

For more information on implementation fidelity outcomes and their scale ratings, please see Supplementary file 1.

##### Community readiness

To further understand how worksite context could potentially influence implementation quality, we conducted employee community readiness phone interviews (n = 125; Supplementary file 2) guided by the Community Readiness Model [37]. Community readiness phone interviewers were structured interviews to identify readiness to change as it related to ‘sedentary time in the workplace.’ We assessed the six domains of readiness (existing efforts, knowledge of efforts, knowledge of the issue, leadership, climate and resources) identified within the Community Readiness Model. These domains are key factors that may influence the workplace’s preparedness to take action on sedentary time in the workplace. We conducted community readiness phone interviews with up to six employees across organization levels from each worksite. Two independent researchers transcribed and scored the interviews using community readiness guidelines [38]. Each researcher read the interviews in its entirety and referred to the Community Readiness Handbook [37] to read the scoring criteria for each domain. Specific statements from the interviews were pulled that reflected a score based on the anchored rating scales for scoring each domain (e.g., existing community efforts scored between 1 = no awareness of the need for efforts to address the issue to 9 = evaluation plans are routinely used to test effectiveness of many different efforts and the results are being used to make changes and improvements). An overall community readiness score was the average score across the six domains ranging from 1 to 9, with 1 = no awareness and 9 = high level of community ownership.

#### i-PARIHS evaluation

Our implementation fidelity outcomes were guided by the i-PARIHS framework. Specifically, we categorized our outcomes into the i-PARIHS constructs of innovation, recipient, and context for potential inclusion in the QCA (Table 1; Supplementary file 1). Categorizing our outcomes by these constructs allow for better interpretability of study implications and increase the ability for study findings to be generalized across other studies.

### Analysis

**Fig. 1:**
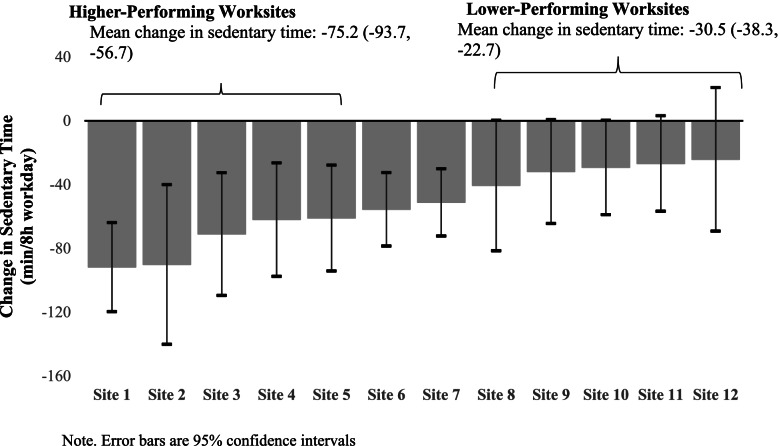
Magnitude of change in sedentary time by worksite

We ranked the 12 worksites in the *STAND* + intervention arm according to the magnitude of change in sedentary time in Fig. 1 [13]. We categorized the top six worksites with the highest magnitude of change as “higher-performing” worksites and the bottom six worksites with the lowest magnitude of change as “lower-performing” worksites. The middle two worksites from our analysis were dropped given the magnitude of change in sedentary time was similar. We generated a code book with the 44 identified conditions (i.e., variables; Table 1) and created decision rules based on i-PARIHS hypotheses related to contextual factors that represent successful implementation, research team experience supporting the implementation of *SMW,* and the intervention factors hypothesized to contribute to the magnitude of change in sedentary time [30]. These decision rules, reported in the results section, were then used for calibration to examine potentially promising variables of interest. A crisp-set QCA was created with dichotomized versions of variables of interest (i.e., present or not present) to allow for an initial reduction of factors that showed promise as necessary and sufficient conditions (i.e., have some pattern of variability across sites). Following calibration, we constructed a truth table to analyze any combinations of the conditions to determine the necessary and sufficient conditions or combination of conditions that increase the likelihood of successful changes in worksite sedentary time. Worksites with missing data for a condition were dropped from the analysis for that condition.

## Results

By design, there was a significant difference in magnitude of change in sedentary time over 12-months between the higher- and lower-performing worksites (mean difference = -44.6, 95% CI = -61.3, -27.9; Fig. 1). Table 1 outlines the decision rules that were developed to examine the presence or absence of a given variable. Eleven promising factors were identified based on having high presence in high performing sites and are outlined in the truth table (Table 2). Promising factors included 4 innovation specific constructs (indoor/outdoor route accessibility; signage visibility; and completion of the advocate survey), 2 context specific constructs (culture supports breaks; morale for the program), and 5 recipient constructs with two focused on advocates (interaction with employees; willingness to continue role) and three focused on employee recipients (face-to-face interactions; walked in a meeting; used the stairs).

**Table 2:** Truth Table

**Worksite**	**Change in sed time**	**Innovation**				**Context**		**Recipient**				
		**Indoor walking route accessibility**	**Outdoor walking route accessibility**	**Communal signage visibility**	**Completed advocate survey**	**Worksite culture supported breaks**	**Perceived morale for the program**	**Advocate's interaction with employees**	**Advocate's willingness to continue role**	**Walked in a meeting**	**Used face-to-face interaction**	**Used the stairs**
**Higher-Performing**												
Site 1	-91.755	**1**	**1**	1	**1**	**1**	0	1	**1**	1	**1**	**1**
Site 2	-90.105	**1**	**1**	0	**1**	**1**	1	1	**1**	1	**1**	**1**
Site 3	-71.049	**1**	**1**	1	**1**	**1**	1	1	**1**	1	**1**	**1**
Site 4	-61.965	**1**	**1**	1	**1**	**1**	0	1	**1**	0	**1**	**1**
Site 5	-61.021	**1**	**1**	1	**1**	**1**	1	0	**1**	1	**1**	**1**
**Lower-Performing**												
Site 8	-40.566	0	**1**	0	**1**	**1**	0	1	**1**	1	**1**	**1**
Site 9	-31.874	-	-	-	0	0	0	-	-	1	**1**	**1**
Site 10	-29.255	**1**	**1**	1	**1**	**1**	0	1	0	1	**1**	**1**
Site 11	-26.826	**1**	**1**	1	0	**1**	0	0	**1**	1	**1**	0
Site 12	-24.215	**1**	**1**	1	**1**	0	0	0	**1**	1	0	**1**
		Necessary	Necessary		Necessary	Necessary			Necessary		Necessary	Necessary

**Bold** numbers represent the necessary conditions for the outcome

*Sed* Sedentary

Of the 11 promising conditions, 7 were considered necessary as they appeared in all higher- performing worksites, but also in some lower-performing worksites. Higher-performing worksites tended to have more accessible indoor (range: 3–5 vs. 2–5) and outdoor (range: 3–5 vs. 3–5) walking routes, more advocate surveys completed (range: 60–100% vs. 20–100%), a culture that supported breaks (range: 65–93% vs. 50–92%), had advocates willing to continue their role (range: 1–3 vs. 1–5), used face-to-face meetings, and used stairs more often compared to lower-performing worksites. Of those conditions, 3 were innovation specific (indoor/outdoor walking route accessibility; completion of advocate survey), 1 was context specific (culture supports breaks), and 3 were recipient specific with focused on the advocate (willingness to continue role) and 2 focused on the employee (use of face-to-face meetings; use of stairs). When examining the pattern of results in the truth table (Table 2), there were no clear patterns of factors that were sufficient to be characterized as a higher- performing site. However, close examination showed that the 2 higher-performing sites and the 2 lower-performing sites were distinguished by the advocates interaction level with employees.

## Discussion

The purpose of this study was to use QCA to examine how study implementation, comprised of intervention fidelity and advocate competence, impacted reduced sedentary time among the higher- and lower-performing intervention worksites. Overall, conditions related to adherence to protocol and the competence of delivery personnel across the i-PARIHS constructs of innovation, recipient, context, were considered necessary but not sufficient as they were present in all higher-performing worksites and some lower-performing worksites. This study provides a novel and unique contribution to the literature given limited knowledge regarding the ability to translate evidence-based workplace sedentary time reduction interventions.

Higher-performing worksites more often provided accessible indoor/outdoor walking routes and promoted a culture that supported informal hourly breaks. This demonstrates the importance of the *SMW* intervention components that focused on environmental and contextual organizational factors including policies and support for enacting such policies to promote a less sedentary workplace. In addition, *SMW* intervention components that facilitated employee engagement with the advocate appeared to lead to superior intervention outcomes. These findings align with the hypotheses that characteristics of innovation (i.e., the *SMW* intervention) contribute to the success of the program, but also suggests that not all characteristics matter equally. Future research in this area should continue to look for the active ingredients of intervention success to increase the likelihood that these ingredients are implemented with high fidelity while also looking for intervention ingredients that may not contribute to outcomes and could potentially be removed from the intervention [39].

Our study also supports that the characteristics of those implementing worksite wellness programs matter. Harvey and Kitson argue that the people who aid in program implementation (i.e., advocates) significantly affect how the program is implemented [27]. More specifically, how they interact with those receiving the intervention and how well-informed they are of the intervention and/or individual(s) receiving the intervention can influence the ease of introducing and sustaining an intervention. From this study, advocates from higher-performing worksites were more willing to continue their role as delivery personnel and completed more advocate surveys (i.e., check-ins). These findings suggest that although program context may influence how programs are delivered [25], the individual(s) delivering the intervention and how they implement it are essential. Thus, strategies to ensure quality of the individual implementing the intervention are necessary. First, it is critical to identify potential program implementers who are communicative, interpersonally connected in the worksite, and concerned about worksite wellness to lead the intervention. Additionally, periodically gauging intervention implementers’ interest in continuing in this role is critical for successful implementation. Including facilitators to help guide implementation by advocates may help intervention implementers identify and overcome barriers to program success.

Strengths of the study include the use of the i-PARIHS framework and the inclusion of both adherence and competence measures to reflect implementation fidelity. Past research typically only examines adherence, limiting the full understanding of intervention fidelity. For example, higher-performing sites had lower or similar adherence to protocol in some areas such as walking route signage and posting of other signage—but still had better reductions in sedentary time compared to lower-performing sites. This could be due to the relative value placed on these strategies to reduce sedentary time. It could also be that who delivers the intervention, and their quality are more likely to lead to employee changes than simply adhering to the protocol. An additional key strength is the use of QCA to provide an innovative and systematic approach for understanding implementation outcomes across contexts and its association with intervention effectiveness [40]. Another strength is the inclusion of a diverse set of worksites allowing for greater generalizability of intervention fidelity implications. Nevertheless, this study has limitations that may influence the interpretation of findings. Although there were no differences in any outcomes by worksite size, *SMW* only recruited small worksites (20–60 employees). Therefore, our study may not be generalizable to larger worksites. Future research is needed with larger worksites; however, this will likely require multiple advocates per site which may introduce additional facilitation challenges. Also, we did not assess the extent to which intervention implementers had previous experience implementing worksite wellness initiatives. However, advocates that were motivated and likely had an interest in health were identified by the worksites. Finally, it is unclear as to what contexts may have made implementation more difficult other than what was assessed in the current study. However, the use of advocates allowed for the adaptation of the program to overcome any contextual challenges. Finally, all of the worksites included in this study significantly reduced sedentary time among their employees which results in our comparison being, potentially about distinguishing between moderate- and higher- performing sites rather than lower- and higher-performing sites. It may be that greater variability in reductions in sedentary time by worksite is needed to get a clearer picture of necessary and sufficient implementation conditions. Some evidence for this weakness includes our finding of a sufficient condition (advocate-employee interaction) that distinguished between the top two and bottom two performing sites.

## Conclusion

Our findings indicate that worksites with high levels of implementation fidelity (i.e., adherence and competence) of the *SMW* intervention resulted in greater magnitude of change in sedentary time compared with worksites that delivered *SMW* with low implementation fidelity. These findings suggest that workplace sedentary reduction interventions may not be implemented consistently and at a high level of quality across worksites, arguably influencing the magnitude of change in sedentary time. Moreover, these findings illustrate the need for future research to identify what factors may influence intervention fidelity, and in turn, effectiveness. For larger scale dissemination of interventions to be effective, researchers need to understand the processes required to implement the intervention consistently and at a high level of quality, especially when different practitioners with different levels of expertise are implementing the intervention in different contexts. Research that advances our understanding of the processes needed to maintain implementation fidelity will be a critical step toward creating sustainable workplace interventions.

## Supplementary Information

**Additional file 1:Supplementary Table 1.** Fidelity measures.

**Additional file 2.** Community Readiness Interview Guide.

## Data Availability

The datasets used during the current study are available from the corresponding author, Dr. Krista Leonard (ORCID 0000–0003-2225–7846), on reasonable request.

## Abbreviation

SMW: Stand and Move at Work
