# Supplementary material for: Qualitative comparative analysis of the implementation fidelity of a workplace sedentary reduction intervention
Source: BMC Public Health. 2022 May 31;22:1086. doi: 10.1186/s12889-022-13476-3 (PMC9158295; doi:10.1186/s12889-022-13476-3)
Supplement: Supplementary file 1 — Additional file 1:Supplementary Table 1. Fidelity measures. [file 12889_2022_13476_MOESM1_ESM.docx]

| Supplementary Table 1. Fidelity measures | | | |  |  |  |  |  |  |
| --- | --- | --- | --- | --- | --- | --- | --- | --- | --- |
| **Advocate survey [*N* = 29]** | | | |  |  |  |  |  |  |
| 1 | ⭘ | Walking route signage visibility | | __/5 | 16 | ▲ | Advocate's interaction with employees | | ___% |
| 2 | ⭘ | Walking route accessibility | | __/5 | 17 | ▲ | Knowledge of employees | | ___% |
| 3 | ⭘ | Communal signage visibility | | __/5 | 18 | ▲ | Advocate's self-efficacy in role | | ___% |
| 4 | ⭘ | Individual signage visibility | | __/5 | 19 | ▲ | Advocate's willingness to continue role | | __/7 |
| 5 | ⭘ | Stair signage visibility | | __/5 | 20 | ▲ | Time spent in the last quarter | | __/5 |
| 6 | ⭘ | Optional strategies chosen** | | __/15 | 21 | ▲ | Time willing to spend in role next quarter | | __/5 |
|  |  |  | # of cultural items (8) |  |  |  |  |  |  |
|  |  |  | # of environmental items (4) |  |  |  |  |  |  |
|  |  |  | # of social items (3) |  |  |  |  |  |  |
| **Participant survey [*N* = 190]** | | | | | | | | |  |
| 7 | ⬛ | Worksite culture supported breaks | | Yes/No | 22 | ▲ | Employees aware of advocate | | Yes/No |
| 8 | ⬛ | Worksite leadership supported breaks | | Yes/No | 23 | ⬛ | Perceived morale for the program | | __/7 |
| 9 | ⬛ | Months used desk of total | | __/5 | 24 | ▲ | Adopted implementation strategy | | __mean (24a-f) |
|  |  |  |  |  |  |  |  | a) Removed waste bin | Yes/No |
|  |  |  |  |  |  |  |  | b) Removed printer | Yes/No |
|  |  |  |  |  |  |  |  | c) Stood in a meeting | Yes/No |
|  |  |  |  |  |  |  |  | d) Walked in a meeting | Yes/No |
|  |  |  |  |  |  |  |  | e) Used face-to-face interaction | Yes/No |
|  |  |  |  |  |  |  |  | f) Used the stairs | Yes/No |
| **Researcher observation (In-person quarterly & web analytics monthly)** | | | | | | | | |  |
| 10 | ⭘ | Sent e-newsletters | | ___% | 25 | ▲ | Attended at least one group advocate call | | Yes/No |
| 11 | ⭘ | Supported informal hourly breaks | | ___% |  |  |  |  |  |
| 12 | ⭘ | Completed quarterly meeting | | ___% |  |  |  |  |  |
| 13 | ⭘ | Completed advocate survey | | ___% |  |  |  |  |  |
| 14 | ⭘ | Supported email distribution | | ___% |  |  |  |  |  |
| 15 | ⭘ | Completed community readiness interview | | ___% |  |  |  |  |  |
| Adherence score ([sum of items 1-15] x 100) | | | | _______% | Competence score ([sum of items 16-25] x 100) | | | | _______% |
| Fidelity (mean % adherence & competence) | | | | _______% |  |  |  |  |  |
|  |  |  |  |  |  |  |  |  |  |
|  | IPARIHS construct = ⭘ Innovation; ▲ Recipient; ⬛ Context | | | | | | |  |  |
| ** | Calculated for each strategy- Waste/recycling bin relocation, centralized printing, ideaboard, standing height tables, hourly informal breaks, face to face interaction, standing meetings, walking meetings, stretch breaks, communal areas, breaks in long meetingss, comfortable shoes, contests, social groups, lunchtime seminar. | | | | | | | | |
