# Supplementary material for: Qualitative comparative analysis of the implementation fidelity of a workplace sedentary reduction intervention
Source: BMC Public Health. 2022 May 31;22:1086. doi: 10.1186/s12889-022-13476-3 (PMC9158295; doi:10.1186/s12889-022-13476-3)
Supplement: Supplementary file 2 — Additional file 2. Community Readiness Interview Guide. [file 12889_2022_13476_MOESM2_ESM.docx]

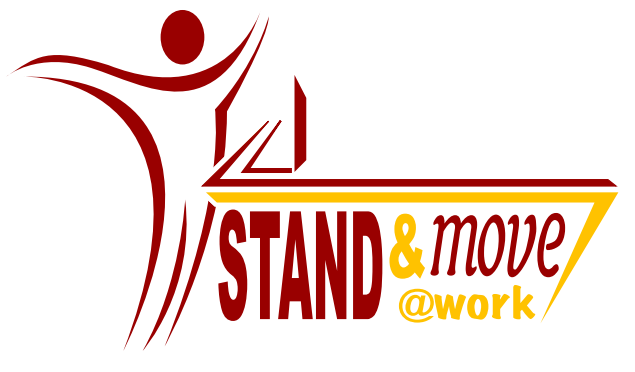


**Community Readiness Interview Guide**

**Date: __________________________________________**

**Worksite of interviewee:_____________________________________**

**Name of Interviewee:______________________________________**

**Name of interviewer:___________________________________**

**Key Informant Interview Questions**

Thank you for taking the time to meet with me today. We will focus on the leadership at the level of the …………………… (worksite name) knowledge about standing more and moving more in the workplace and resources for tackling this issue (people, money, time, space, etc.).

I’d like to start by telling you more about what we mean by “standing more” and “moving more.” In most workplaces individuals spend a lot of their day sitting. There is evidence to suggest that too much sitting could have negative health impacts. “Standing more” refers to when an employee replaces some sitting with standing. This standing could occur at one’s desk, in a meeting, or elsewhere at work. “Moving more” refers to activities where the employee is moving while they are at work. This might be activities such as getting up and walking to speak with their neighbor, taking the stairs instead of the elevator, or having a walking meeting. “Moving more” does not refer to planned activities such as exercise.

To keep things simple, we will be referring collectively to these terms as “standing and moving more.” Do you have any questions about this?

I also want to clarify with you that the questions I’m going to ask refer specifically to your department or unit, and not to the larger organization you are a part of. Please do your best to answer the questions as it pertains to your specific workplace.

Also, you may notice that some of these questions seem repetitive. This is part of the interview process. Please do your best to repeat your answers for each question.

Do you have any questions before we begin?

**JOB STATUS**

1. Can you please state your job position?
   1. Please tell me a little about your job duties?
      1. **PROMPT:** What types of activities do you do on a daily basis?
   2. How many years have you been with the company?
   3. How many years have you been in your current position?

**LEADERSHIP**

- **"Leadership" can include anyone within your workplace who is appointed to a leadership position or is influential in community affairs, i.e., a Manager, CEO, Team leader, Administrator, Director etc.**

1. Who is your supervisor? What role do they play in the worksite?
   1. If talking with “leader” or worksite, then ask what role they play in the worksite (i.e. what is happening on their level)
2. Who, in your opinion, are the informal leaders at your workplace?
   - 1. **PROMPT**: Whose opinion is respected and/or are influential or who may be contacted when issues arise?
   1. 3a. How did this person (these people) become the "leader(s)"?
   2. 3b. Does this person advocate for employees needs/concerns related to worksite wellness?
3. Who would an employee talk to first if he or she were concerned about worksite wellness?
   1. 4a. What position does this person have?
      1. **PROMPT:** Why would you talk to the person in this position?

**WORKPLACE EFFORTS TO INCREASE STANDING AND MOVING**

1. Can you describe any workplace wellness initiatives, if any, that have been made or are being made? (these can include programs regarding nutrition, exercise, sleep, etc)
2. Please describe the efforts or activities, if any, that have been made or are being made to help you stand or move more at work.
   - 1. ***If answered no or don’t know, then skip to question 7.***
   1. 6a. How long have these efforts been going on in your workplace?
   2. 6b. What do you think are the strengths of these efforts?
   3. 6c. What do you think are the weaknesses of these efforts?
   4. 6d. How are these efforts being evaluated?
      1. ***If answered no or do not know to question, then skip to question 7.***
   5. 6e. What are the evaluation results being used for?
3. Do you think the leadership would support (any/additional) **efforts** to standing and moving more at work?
   - 1. ***If yes, proceed to 7a. If no, proceed to 7b and 7c.***
   1. 7a. If you think they ***would*** support efforts, then what sort of support would they provide?
      1. **PROMPT:** What do you think motivates them to support standing and moving more?
      2. ***If they support efforts skip to question 8.***
   2. 7b. If you think they ***wouldn’t*** support efforts, then why not?
   3. 7c. If you think they ***wouldn’t*** support efforts, then what do you think would need to happen in order for them to consider standing and moving more at work?

**KNOWLEDGE ABOUT WORKPLACE EFFORTS**

1. On a scale from 1 to 10, with 1 being “unaware” and 10 being “very aware” how aware are people in your workplace about the efforts being made to stand and move more ?
   - 1. **PROMPT:** Why did you give this a rating of __________?
     2. ***If answered 0 skip to question 11***
2. On a scale of 1 to 10, with 1 being “not at all” to 10 being “a very great concern”, how does the leadership rate excess sitting as a problem in your workplace?
   - 1. **PROMPT:** Why did you give this a rating of ____________?
3. On a scale of 1 to 10 with 1 being “not at all” to 10 being “definitely yes”, how much does the leadership view sitting at your desk a *desirable* component of the workplace?
   - 1. **PROMPT:** Why did you give this a rating of ____________?
4. Do you think the leadership sees any benefits in having employees take a break from their desk to stand and move more at work?
   - 1. **PROMPT:** What are the benefits that they see?
5. Do you think the leadership sees any disadvantages in having employees take a break from their desk to stand and move more at work?
   - 1. **PROMPT:** What are the disadvantages that they see?
6. Is there a group or committee that has been formed to discuss workplace wellness?
   - 1. ***If yes, proceed to 13a and 13b, if no proceed to number 14.***
   1. 13a. Which leaders are involved in these efforts?
   2. 13b. Has this group or committee discussed standing and moving more at work?
7. Do you think the leadership in your workplace community would support **changes in** **policy** related to standing and moving more at work?
   - 1. **PROMPT**: Does the leadership think that it should have a role in reducing excess sitting at work (and promoting standing and moving more at work)?

**COMMUNITY CLIMATE**

1. How does the workplace community (e.g., wellness coordinators, employee committees, coworkers) support the efforts to stand and move more in your workplace?
2. What do you think are the primary obstacles that the workplace community faces in promoting standing and moving more at work?

**KNOWLEDGE ABOUT THE PROBLEM**

1. What is your personal knowledge about sitting at work and your health?
   1. 17a.How did you gain this knowledge?
2. Do you think that employees in your workplace have personal knowledge about sitting at work and their health?
   1. 18a. **WHY** do you think this is?

**RESOURCES FOR PREVENTION EFFORTS**

1. Who would a concerned employee turn to first for advice regarding excess sitting at work?
2. Who in your workplace community provides resources for the efforts you listed earlier?
3. What is the level of expertise and training among those in your workplace who are working toward increasing standing and moving in the workplace?
4. Do you have a designated wellness coordinator?
   - 1. **PROMPT:** What is their background? Qualifications?
5. What is the workplace attitude about supporting efforts to stand and move more at work using the resources of **staff, money, time and/or space**?
   - 1. **PROMPT:** Provide clarification on the meaning of resources as related to staff, money, time, and/or space. Only to make sure that the interviewee has had enough time to answer with respect to **staff, money, time, and/or space.**
